# On the relaxation time of interacting superparamagnetic nanoparticles and implications for magnetic fluid hyperthermia

**DOI:** 10.3762/bjnano.10.127

**Published:** 2019-06-24

**Authors:** Andrei Kuncser, Nicusor Iacob, Victor E Kuncser

**Affiliations:** 1 National Institute of Materials Physics, P.O. Box MG 7, 077125, Magurele, Romania

**Keywords:** magnetic hyperthermia, magnetic nanoparticles, magnetic relaxation time, micromagnetic simulation

## Abstract

A critical discussion on the presently available models for the relaxation time of magnetic nanoparticles approaching the superparamagnetic regime in the presence of interparticle dipolar interactions is considered. The direct implications of such interactions for magnetic fluid hyperthermia therapy through susceptibility loss mechanisms give rise to an indirect method for their study via specific absorption rate measurements performed on ferrofluids of different volume fractions. The theoretical support for the specific evolution of the relaxation time constant and the anisotropy energy barrier versus the interparticle interactions in a perturbation approach of the simple Néel expression for the relaxation time is provided via static and time-dependent micromagnetic simulations.

## Introduction

Magnetic relaxation phenomena in nanoparticulate systems are under intensive investigation today, especially due to their implications in various fields of nanotechnology such as biomedicine, magnetic data storage and sensors [1–6]. Concerning the biomedical applications, the magnetic relaxation of nanoparticles is of key interest in magnetic resonance imaging (through the influence of the relaxation time of the nanoparticulate contrasting agents on proton relaxivity [7–9]) and cancer therapy (through magnetic fluid hyperthermia therapy [10–11]). The efficiency of the magnetic nanoparticles (MNPs) in a colloidal system to convert the energy of AC magnetic fields into temperature increments is of high importance for magnetic hyperthermia therapy. Usually the system consists of MNPs dispersed in an aqueous medium, known also as a ferrofluid. The heat transfer from the specifically configured AC field (with biologically compatible amplitude and frequency) to the tissue loaded with suitably functionalized MNPs can be performed by different mechanisms, depending on type and size of the MNPs. In the case of magnetic oxide nanoparticles (usually ferrites) with an average size of less than 30 nm (considered at this moment as the most suitable for such purposes), two heat transfer mechanisms must be considered [12–13]: (i) hysteretic losses and (ii) magnetic relaxation processes. The effective mechanism depends on the relationship between the magnetic relaxation time, τ, and the inverse of the AC field frequency, 1/*f*, defining the time window, τ_M_, of the magnetic excitation (τ_M_ may also represent a measuring time window). In this respect, two regimes have to be mentioned here [14]: (i) a static regime, corresponding to τ >> τ_M_, where the main heat transfer mechanism is through hysteretic losses and (ii) the dynamic regime, corresponding to τ << τ_M_, where the main heat transfer mechanism is through magnetic relaxation phenomena. The relaxation time depends on the volume of the MNPs, and in the case of nanoparticulate systems of finite size, also on the polydispersity. Both mechanisms can contribute, with the first, for nanoparticles larger than a critical size, and the second, for finer nanoparticles. It is thus very important that both the size distribution of the MNPs and the relaxation time corresponding to each volume are well characterized.

The heat transfer is reflected in a power deposition term, *q*_p_. Among other parameters, this term depends on the volume fraction of the nanoparticles in the ferrofluid, φ, which is defined as φ = *V*_MNPs_/(*V*_MNPs_ + *V*_FF_) where *V*_MNPs_ is the total volume of the MNPs and *V*_FF_ is the total volume of the ferrofluid. In turn, *q*_p_, which quantifies the energy transfer from the magnetic AC field to the ferrofluid (or specific tissue loaded by MNPs), is related to the specific absorption rate (SAR). Actually the SAR represents the power absorbed per mass unit of ferrofluid (W/kg). Accordingly, SAR = *P*/*m* = *c* × Δ*T*/Δ*t* = *q*_p_/ρ, where *P* is the power dissipated in the mass *m* of ferrofluid. Terms *c* and ρ are the specific heat and the density of the ferrofluid respectively whereas Δ*T*/Δ*t* is the rate of the temperature increment. The above relation is valid only for adiabatic systems where the absorbed energy is completely transformed into heat. The SAR can be simply evaluated if the rate of the temperature increment is experimentally estimated in adiabatic-like systems. In order to reach such conditions, the experiments have to be done on low volume ferrofluid systems (simulating as much as possible the loaded tissue) with suitable isolation aimed to diminish the heat losses. However, even extremely careful experimental caution cannot completely prevent the heat transfer from the actuated ferrofluid. Therefore, new methodologies to properly compensate for the heat losses have been proposed over time. The simplest procedure of taking the initial heating slope [11,15] has been extended to more sophisticated cases of correcting the heating curves through suitably exploited cooling curves [16–17] or alternative calorimetric calibrations [18]. Once an experimental methodology for a suitable estimation of the SAR is reasonably achieved, two opportunities appear: (i) the possibility of building a SAR database for nanoparticulate systems obtained by different processing routes as a function of particle size, volume fraction and dispersing liquid parameters and (ii) the possibility to investigate the mechanisms involved in the heat transfer under the AC field actuation as thoroughly as possible. The first option is directly connected to realistic numerical solutions of the bio-heat-transfer equation which is of key interest in the optimization of cancer therapy by magnetic hyperthermia therapy [19]. The second option may be effective in exploiting the theoretical models proposed for linking the experimental SAR dependencies to the physical mechanisms. From the theoretical point-of-view, the heat transfer is only due to the actuated nanoparticles, and therefore, the power, *P*, absorbed by the ferrofluid is in fact the power absorbed by the nanoparticles. Accordingly, an additional coefficient SAR_NP_ counting for the power dissipated by the mass of the nanoparticles can be defined, the link between the two coefficients being expressed as:

[1]
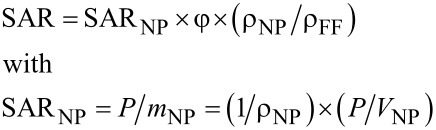


In the above relation, *P*/*V*_NP_ = P* represents the absorbed power per unit volume of MNPs which can be directly related to the evolution of the magnetization (or susceptibility) of the system (magnetic moment per unit volume).

For nanoparticles in the static regime, P* can be simply expressed by multiplying the area of the hysteresis loop developed under the amplitude of the AC field with its frequency. However, according to Equation 1, the experimentally obtained SAR values should increase linearly with φ under the condition that P* does not depend explicitly on φ. However, both experimental evidence and Monte Carlo modeling have been provided in [20] to understand the influence of the dipolar interparticle interactions on P*, leading to a reduction of the heating power and consequently on the hyperthermia effects of ferromagnetic nanoparticles.

For noninteracting MNPs in the dynamic magnetic regime (superparamagnetic regime), it has been shown by Rosensweig in 2002 [21] that only a susceptibility loss mechanism has to be considered (P* ≈ χ’’ where χ’’ is the out of phase component of the magnetic susceptibility). Furthermore, earlier more sophisticated theories have been considered in order to investigate the role of the interparticle interactions on the magnetic relaxation phenomena or on the behavior of the magnetic susceptibility. The most successful of these approaches is related to the first order and second order modified mean field theory [22–23]. It should be noted that the mean field theory (providing means to include the effective field experienced by a particle due to the action of the neighboring nanoparticles) at various level of approximation has been tested with respect to experimental dependencies. This led to available approaches for describing the more complex cases related to particle size distribution and different concentrations. The influence of the dipolar interactions on the frequency-dependent magnetic susceptibility has been more recently extensively studied by Ivanov et al. [24–25]. For example, in [25] the Debye theory of polar relaxation was applied in the case of MNPs undergoing only Brownian motion. This was further extended to a first order modified mean field theory leading to a relatively simple expression of the out-of-phase component of the susceptibility in ferrofluids with dipolar interparticle interactions. Accordingly, the out-of-plane component of the susceptibility for interacting MNPs is proportional to the out-of-phase component of susceptibility of similar noninteracting MNPs modulated by a factor which depends on the in phase component of susceptibility of noninteracting MNPs. In spite of its simplicity, this approach cannot be easily applied to hyperthermia applications, where the nanoparticles are usually vectorially bound to the tissue and Brownian relaxation can be neglected. However, even in the case of usual ferrofluids consisting of dispersed and motion-free nanoparticles, the Brownian relaxation mechanism becomes dominant over the Néel mechanism only for nanoparticles larger than a critical radius (e.g., 8 nm in the case of magnetite nanoparticles) [21]. In such conditions, the heat transfer mechanism might also be completed by a hysteretic loss (the nanoparticle size can be large enough to open a hysteresis loop), so it becomes really difficult to compare experimental results on SAR with the above-mentioned approach. On the other hand, according to the above-mentioned theory, the out-of-phase component of the susceptibility of interacting nanoparticles can be expressed only as a function of components of noninteracting nanoparticles, which gives support for the validity of a perturbational approach towards an upper limit of volume fraction, with the possibility to extend such reasoning to the expression of the relaxation time. In fact, the first experimental support for the specific influence of the magnetic dipolar interactions on different parameters in the relaxation law of nanoparticles in the dynamic magnetic regime has been provided in [26]. Accordingly, it has been experimentally proven that the effect of the nanoparticle interaction is not only to increase the anisotropy energy barrier per nanoparticle but also to decrease the specific time constant in the simplest Néel–Brown expression of the relaxation time, which is usually considered only as a material dependent parameter. In this context, the threefold aim of this paper is: (i) to critically discuss the presently available models for the relaxation time of nanoparticles in the magnetic dynamic regime with focus on the effect of the interparticle interactions, (ii) to provide a specific approach for the experimental investigation of the interparticle interactions through SAR measurements and (iii) to provide theoretical support for the specific evolution of the relaxation time constant and the anisotropy energy barrier in the Néel expression as function of interparticle interaction through a suitable exploitation of static and time-dependent micromagnetic simulations.

## Results and Discussion

### Relaxation time and interparticle interactions: a critical survey

The first expression of the relaxation time of magnetic monodomain particles (of Stoner–Wohlfarth-type) with the thermal excitation energy (*kT*) approaching the particle anisotropy barrier energy (e.g., Δ*E* = *KV* in the case of uniaxial anisotropy) has been provided by Néel under the assumption that the particle macrospin behaves as a gyroscopic system [27]:

[2]
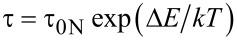


In Equation 2, τ_0N_ is a time characteristic depending slightly on temperature and other material parameters such as magnetization, gyromagnetic ratio, Young’s modulus, etc. An improved model for the magnetic relaxation was performed by Brown who supposed that the orientation of the particle macrospin may be described by the Gilbert equation. Accordingly, the relaxation time was introduced as a specific parameter in a Fokker–Planck-type equation devoted to the probability density of orientation [28]. Brown derived different formula for the relaxation time (in different approximations for the Δ*E*/*kT* ratio) which were further refined by Aharoni [29]. However, their form did not significantly differ from Equation 2, the only difference being in the expression of the time factor τ_0_ (hence the general name of Néel–Brown for Equation 2). Other expressions of the relaxation time have been further obtained by solving the Fokker–Planck equations in different approximations, as excellently summarized in [30]. As a general behavior, the exponential dependence of the relaxation time versus the ratio Δ*E*/*kT* remains valid as soon as this ratio is higher than unity.

Different attempts to consider the influence of the interparticle dipolar interactions on the relaxation mechanisms have been provided by Mørup et al. [31–32] and Dormann et al. [30]. The first idea in a perturbation theory due to a mean field effect of the dipolar interactions is to express the magnetic energy of the system by a superposition of uniparticle energies, with the anisotropy energy modified by an additional Zeeman term due to the presence of the particle magnetic moment in the effective field created by the neighboring particles. Such an approach has been used in [30], resulting in the relaxation time (Equation 2) in the new corrected form:

[3]
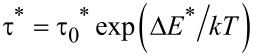


with ΔE* > Δ*E* (e.g., Δ*E* = *KV* for uniaxial symmetry and lack of interparticle interactions) and τ_0_* < τ_0_ (e.g., τ_0_ is the relaxation time constant for noninteracting MNPs), where both starred (*) parameters depend on the number and arrangement of the neighboring MNPs. According to Dormann’s model, the interparticle dipolar interaction will always increase the anisotropy energy barrier and will decrease the time constant with respect to the case of noninteracting particles, but in a such a way that the relaxation time will remain larger in the first case. On the contrary, Mørup et al. [31] have reported, starting from temperature-dependent Mössbauer spectroscopy data obtained on maghemite based ferrofluids, a decrease of the relaxation time with increasing volume fraction. An intriguing explanation based on the decrease of the anisotropy energy per nanoparticle due to the dipolar interactions for the specific *kT*/*KV* ratio has been provided in a subsequent paper [32]. As a consequence, the effect of the interparticle interactions on the magnetic relaxation time, which is of key importance in magnetic hyperthermia applications, has remained an open issue to be investigated both experimentally and theoretically.

### Experimental investigation of the relaxation time through SAR measurements

According to the above-mentioned issues, the proposed approach for the investigation of the relaxation time through SAR measurements is to express the power absorbed per unit volume of nanoparticles, P*, within Rosensweig’s model [21] specific to monodisperse noninteracting nanoparticles of a given volume, *V*. Nanoparticles that are smaller than a critical size that lead to the predominance of the Néel relaxation mechanism over the Brownian one are considered. Taking into account only a perturbative effect of the interparticle interactions on the magnetic relaxation mechanism, it will be implicitly assumed that the above model may be used up for an upper limit of volume fraction, φ, with the only effect of the perturbation reflected in the expression of the relaxation time. Accordingly, the dissipated power is

[4]
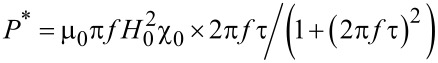


where τ is the effective relaxation time (only the Néel component), *f* and *H* are the frequency and the amplitude of the applied AC magnetic field, µ_0_ is the permittivity of air and χ_0_ is the equilibrium susceptibility (χ_0_ 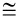 µ_0_η*M*_s_^2^*V*_M_/3*k*_B_*T* where *M*_s_ and *V*_M_ are the spontaneous magnetization and the magnetic volume of the nanoparticle, respectively). The simple Néel–Brown expression,

[5]
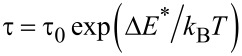


was considered, where τ_0_ is a time constant (supposed to depend only on the material, because the temperature variation in hyperthermia is of only a few degrees) and ΔE* is an anisotropy energy barrier (supposed to be modified by interparticle interactions).

According to the Equation 1 and the definition of SAR(*T*), we find

[6]
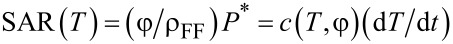


**Figure 1:**
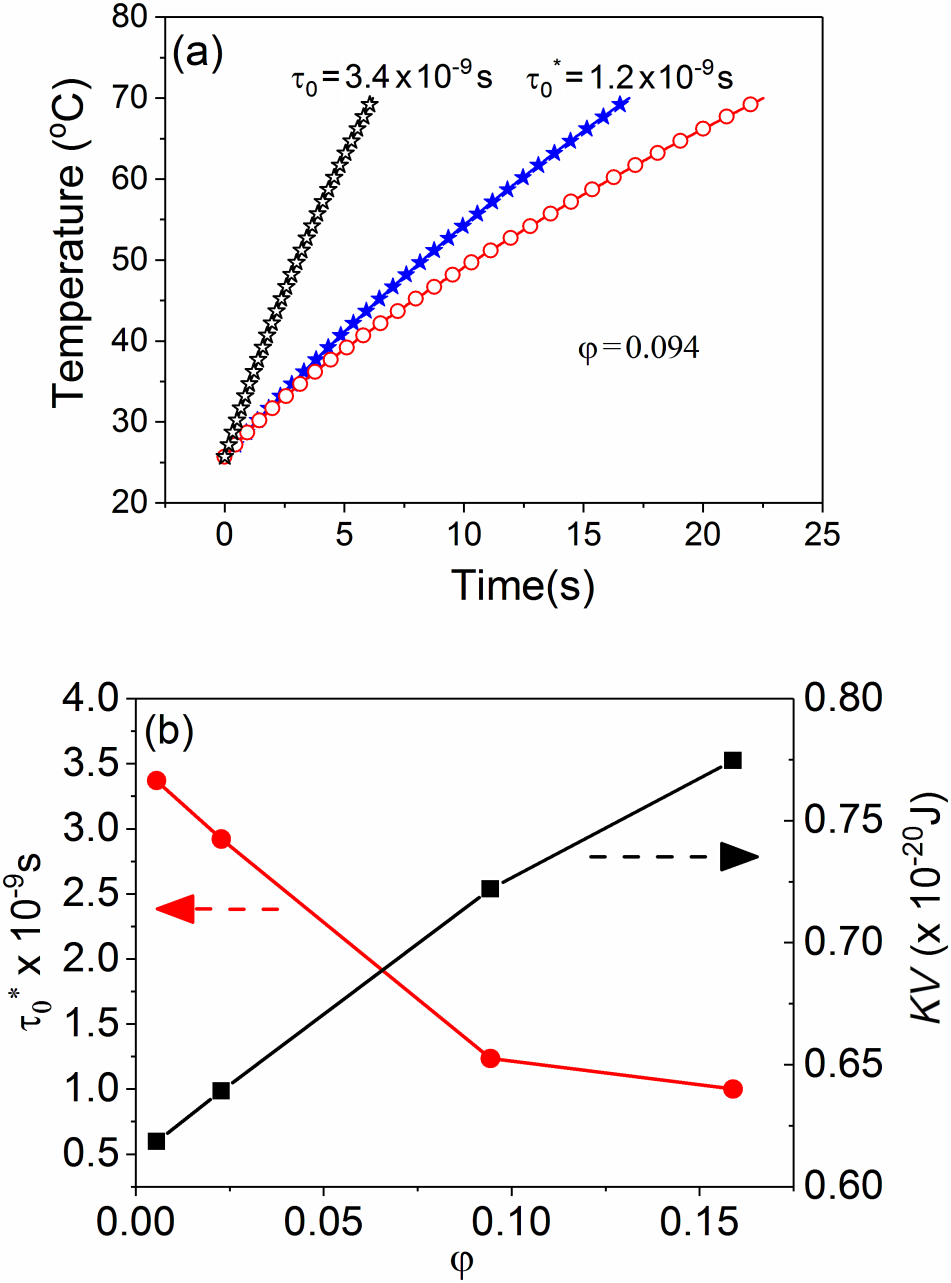
(a) Red open circles – adiabatic curve obtained by a proper experimental methodology in the case of a ferrofluid with φ = 0.094, black open stars – theoretical curve obtained from the Rosensweig model with input parameters specific to the ferrofluid with φ = 0.094 (e.g., *KV* = 7.2 × 10^−20^ and *H*_0_ = 21 kA/m), but considering for the relaxation time constant the value obtained from a more diluted sample (τ_0_ = 3.4 × 10^−9^ s, φ = 0.005), filled blue stars – the same theoretical curve adjusted to τ_0_ at 1.2 × 10^−9^ s for the best fit of the adiabatic curve. (b) Volume fraction dependence of τ_0_* and anisotropy energy barrier (ΔE*) as experimentally obtained.

The time increment of the ferrofluid temperature, *T*(*t*), can be evaluated versus parameters specific to P* by the numerical integration of the last part of Equation 6. On the other hand, *T*(*t*) can be also experimentally obtained by heating the ferrofluid under a completely characterized AC magnetic field and using specific methodologies to minimize the heat losses [16–17]. The investigation of the relaxation mechanism can be therefore performed by the simulation of the experimentally obtained *T*(*t*) curves (adiabatic-like) through the theoretical *T*(*t*) dependence obtained by integration of Equation 6, under the condition that the material-dependent parameters for the magnetite nanoparticle based ferrofluids of different volume fractions were independently derived by alternative techniques [26,33]. Detailed experimental and methodological aspects involving a very diluted ferrofluid as reference are described in [26]. The adiabatic-like curve obtained in the case of a concentrated ferrofluid (φ = 0.094) consisting of quasi-ellipsoidal magnetite nanoparticles of average magnetic volume of 4.3 × 10^−25^ m^3^ dispersed in transformer oil, with a spontaneous magnetization *M*_s_ = 4.5 × 10^5^ A m^−2^, as determined by DC low temperature magnetometry and an effective anisotropy energy barrier ΔE* = 7.2 × 10^−21^ J, as estimated by Mössbauer spectroscopy, is presented in Figure 1 (red open circles). The amplitude of the AC field was 21 kA/m, as numerically estimated from the current amplitude and geometrical characteristics of the RF induction coil, by a finite element method.

Figure 1 also shows two theoretical simulations according to the numerical solution of Equation 6 involving the Rosensweig model for power P* (Equation 4) with the above-mentioned input parameters and for two different time constants: τ_0_ = 3.4 × 10^−9^ s and 1.2 × 10^−9^ s. The first time constant corresponds to the best fit of the experimental curve of a similar system but for a very low volume fraction (φ = 0.005). It can be observed that this value (typical for noninteracting nanoparticles) cannot conveniently fit the experimental curve for the concentrated system (with φ = 0.094), for which the best fit of the initial slope is obtained for a lower value of τ_0_ (1.2 × 10^−9^ s in this case). A direct proof for the dependence of τ_0_ on the strength of the dipolar interactions is provided by the above example.

A more extended experimental study on the influence of the volume fraction and external AC field amplitude on the SAR values has been previously performed and partially reported in [26]. The work was performed on similar ferrofluids with four different volume fractions, where the ferrofluid of the lowest volume fraction (φ = 0.005) is considered as the reference for noninteracting nanoparticles. All samples were magnetically excited by a radiofrequency magnetic field with a constant frequency of 235 kHz (single radiofrequency inductor) and at four amplitude values: 14 kA m^−1^, 21 kA m^−1^, 28 kA m^−1^ and 35 kA m^−1^. As expected, the experimental SAR values increase with the volume fraction (in a more saturated manner as compared to the predicted linear dependence), the increment being larger for higher amplitudes of the AC field (e.g., for 14 kA m^−1^ the SAR increases from 0.3 W/g in the ferrofluid with φ = 0.005 to 2.8 W/g in the ferrofluid with φ = 0.16 whereas for 28 kA m^−1^ the SAR increases from 1.1 W/g in the ferrofluid with φ = 0.005 to 4.0 W/g in the ferrofluid with φ = 0.16). According to the above reported values (expressed in W/g of ferrofluid), the SAR increases with the square of the field amplitude (as theoretically expected) in the case of very diluted ferrofluids, and much more slowly in the case of concentrated ferrofluids (e.g., for φ = 0.16, SAR increases less than two times when the field amplitude doubles). Note that any comparison between the SAR values obtained under different experimental conditions and on different ferrofluids (even formed by a same type of nanoparticles) is not conclusive. In order to avoid the effect of the dilution, the SAR values expressed in W/g of magnetic material can be used. Based on the definition of the volume fraction and taking a density of roughly 5.6 g/cm^3^ for magnetite nanoparticles and 0.9 g/cm^3^ for the transformer oil, one can compute a quantity of 0.03 g of magnetic material in 1 g of ferrofluid in the case of the sample with φ = 0.005 and of 0.54 g of magnetic material in 1 g of ferrofluid in the case of the sample with φ = 0.16. Furthermore, the SAR values of about 37 W/g of magnetic material and 7.5 W/g of magnetic material are deduced for the sample with φ = 0.005 and for the sample with φ = 0.16, respectively (under the same experimental excitations with the field frequency of 235 kHz and the field amplitude of 28 kA m^−1^). Nevertheless, such a strong reduction of the heating power due to only interparticle interactions should be taken into account in hyperthermia biomedical applications, which has also been reported in other previous studies [34–35].

### Theoretical investigation of the relaxation time by micromagnetic simulations

The dynamic magnetic behavior of parallelepiped nanoparticles with specific configurations (reflecting different volume fractions) has been simulated using the object oriented micromagnetic framework [36], an open source software developed by the National Institute of Standards and Technology. The core software uses numerical methods (Euler or Runge–Kutta) in order to solve a system of Landau–Lifshitz–Gilbert (LLG) equations describing the time evolution of each magnetic moment (only one magnetic moment is assigned to each monodomain-like nanoparticle). For the specific purpose of this work, i.e., the observation of the magnetic behavior under thermal excitations, an extension of the program, as developed by Oliver Lemke, was used [36]. The influence of temperature on the particulate magnetic moments, **m**, has been modeled by altering the original LLG equation with a strongly fluctuating term **h_fluct_**. Thus, the LLG equation becomes a Langevin-type stochastic differential equation [28,37]:

[7]
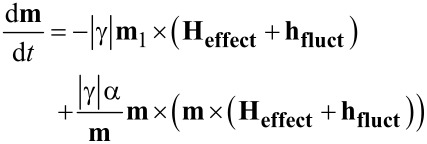


The values of **h_fluct_** at each magnetic moment are provided according to a Gaussian distribution which is characterized by two parameters, the mean and the variance, taking values of 0 and


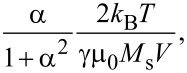


respectively, where γ is the gyro-magnetic ratio and α the damping constant (the usual value of 0.5 was considered). *M*_s_ and *V* are the spontaneous magnetization and the volume of the magnetic material/MNP, respectively, and **H_effect_** is an effective field originating from the total magnetic energy of the system (with anisotropy energy, demagnetization energy, exchange energy and Zeeman energy, as components).

**Figure 2:**
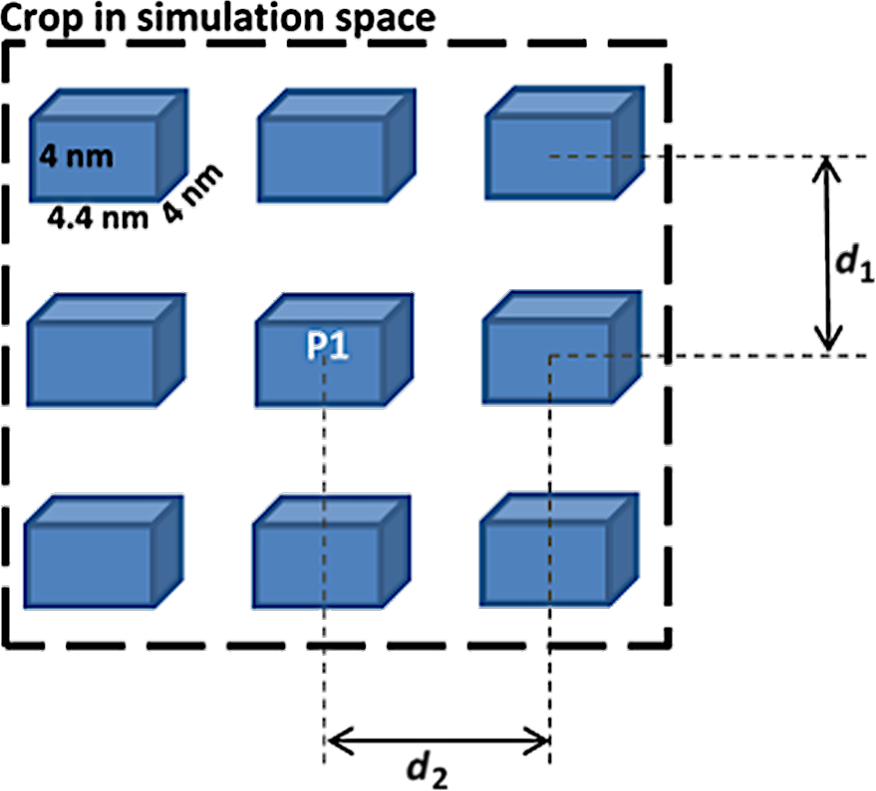
The specific geometry of magnetic nanoparticles in the micromagnetic simulations. Different configurations may be obtained for different couples of *d*_1_ and *d*_2_ distances.

A group of thirteen magnetic nanoparticles (each of size 4.4 × 4 × 4 nm in order to assure the uniaxial magnetic anisotropy) with no magneto-crystalline anisotropy, a stiffness constant (*A*) of 1.3 × 10^−11^ J/m^3^ and a spontaneous magnetization (*M*_s_) of 8.5 × 10^5^ A/m have been arranged in a bidimensional geometry as in Figure 2.

Note that the magnetic parameters of realistic order of magnitude have been chosen in order to reflect real experiments on magnetite and also to assure the magnetic monodomain-like behavior for the nanoparticles. However, the considered nanoparticles differ in size/shape and bidimensional arrangement from those leading to the results reported in Figure 1, and therefore, rather a qualitative agreement should be expected between simulation and experiment. In this particular case, the volume fraction (as a measure of the particle density) will be computed as the volume occupied by touching nanoparticles relative to the total volume occupied by the 13 nanoparticles distributed in only one layer, being noted accordingly by φ*. The particles are considered to be magnetic monodomain and consequently defined by their associated magnetic moment (or cell magnetization), considering a fixed nanoparticle volume in each occupied cell (thus the terms MNPs and magnetic moments will be further used interchangeably). Time-dependent simulations have been performed for three different densities of nanoparticles, reflected by the following interparticle distances *d*_1_ and *d*_2_: high density (volume fraction) with *d*_1_ = 8 nm and *d*_2_ = 8.8 nm (i.e., 1 free cell between neighboring MNPs), medium density with *d*_1_ = 12 nm and *d*_2_ = 13.2 nm (i.e., 2 free cells between neighboring MNPs) and low density with *d*_1_ = 16 nm and *d*_2_ = 17.6 nm (i.e., 3 free cells between neighboring MNPs). For static simulations, a special case was considered, namely a purely noninteracting case of a very low density of MNPs (7 free cells spacing between neighboring MNPs, with *d*_1_ = 32 nm and *d*_2_ = 35.2 nm).

The equivalent volume fraction, φ*, corresponding to the interparticle spacing of *n*_cells_ = 1, 2 and 3 cells, respectively, can be straightforwardly estimated to be 1/(1 + *n*_cells_)^2^, resulting in approximate values of 0.25 for high density packing, 0.11 for medium density packing and 0.06 for low density packing.

Both time-dependent and time-independent simulations have been performed, each of them being briefly discussed with respect to the provided results and limitations.

### Time-independent approach: simulation and data analysis

**Figure 3:**
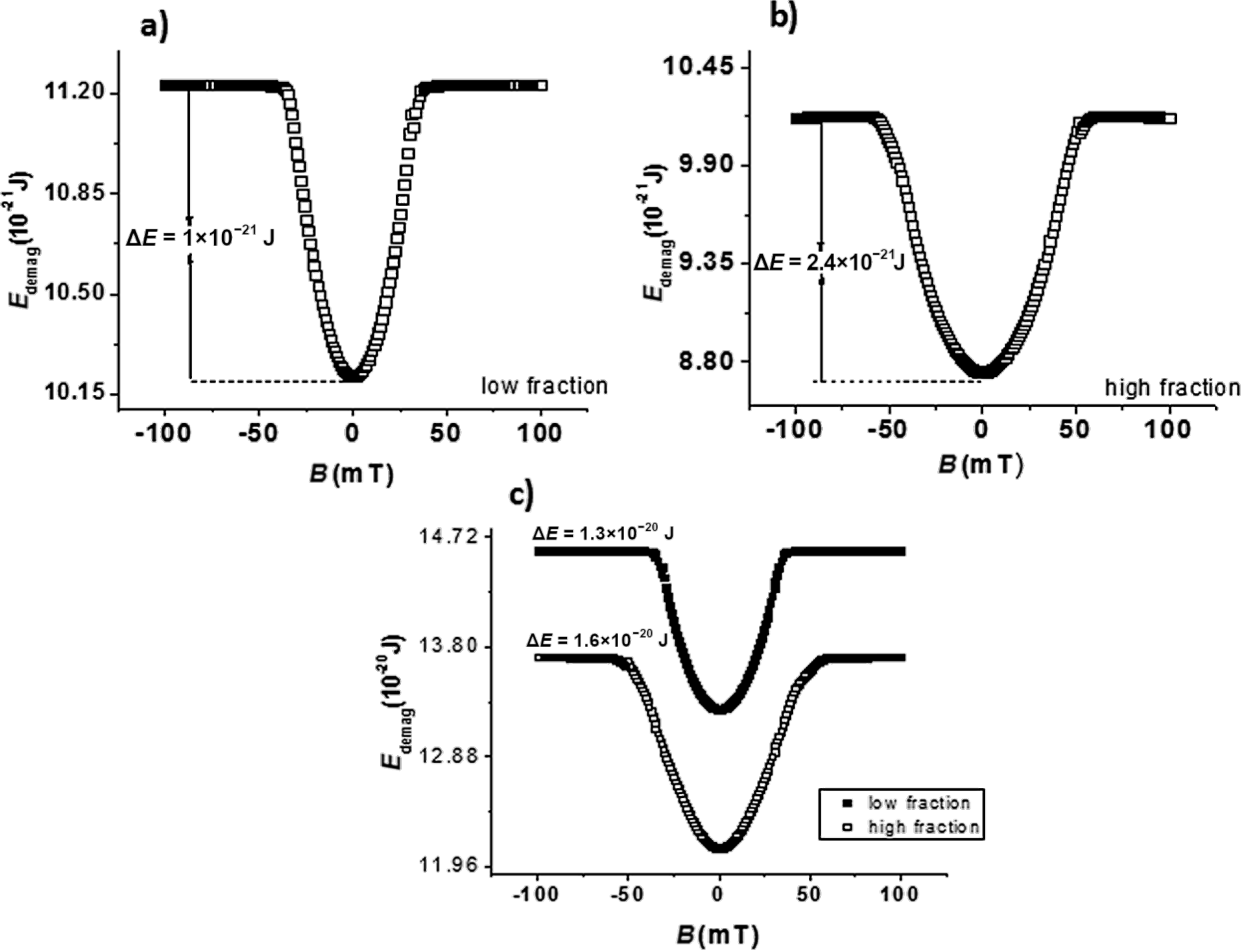
Demagnetization energy profiles and anisotropy energy barriers for MNP P1, for low and high volume fractions, respectively (a and b) and for the whole considered nanoparticulate system for the same configurations (c).

Regarding the time-independent analysis, a monotonously increasing magnetic field ranging from −100 mT up to 100 mT has been applied along the *Oy* axis (perpendicular to the MNP length). Considering the uniaxial anisotropy (as due to only the shape anisotropy), defined along *Ox* axis (along the MNP length), it is possible to calculate both the magnitude of the anisotropy energy barrier of each particle (e.g., as in Figure 3a,b) or the anisotropy energy of the whole system (e.g., as in Figure 3c) for all the assumed three configurations by simply scanning the magnetic field orthogonal to the anisotropy axis and computing the demagnetization energy.

Figure 3a shows the demagnetization energy profiles of particle P1 as function of the applied magnetic field in the case of the low volume fraction configuration. The fact that the energy profiles are independent of the particle surroundings (i.e., number of first order neighbors) gives support for noninteracting particle behavior for the chosen low volume fraction. Another observation that shows the independent nature of the NPs for the low volume fraction case is the relation between the overall potential barrier and the potential barrier of individual particles, i.e., the 1.3 × 10^−20^ J energy barrier (Figure 3c, top) is the sum of 13 one particle energy barriers of 1 × 10^−21^ J (Figure 3a).

On the other hand, Figure 3b and Figure 3c (bottom), corresponding to the case of a high volume fraction, shows a strong influence of the particle surroundings on the particle energy barrier. For example, according to Figure 3c, the energy barrier of MNP P1 is much higher in the case of a high volume fraction configuration as compared to the case of a low volume fraction configuration. Thus, a strong interaction between the nanoparticles of the system can be concluded for this case, leading to an overall energy barrier significantly different from the sum of the energy barriers belonging to each particle in the configuration. Note also that the outer particles, with a less symmetrical surrounding, present a demagnetization energy profile (not shown here) which differs from a Stoner–Wohlfarth-like behavior, in contrast to the case of the inner nanoparticle P1 for which this behavior is roughly respected (see Figure 3b). However, in the real experimental cases dealing with a very high number of MNPs, the inner ones (with a complete number of neighbors) are by far dominant over the outer ones. Therefore, the time-dependent analysis will be performed only for such inner nanoparticles with Stoner–Wohlfarth-like behavior, sensing the interparticle dipolar interactions only as a perturbation of their own anisotropy energy. It is worth mentioning that the demagnetization energy profile of the whole system of 13 particles is also influenced by the volume fraction (and hence by interparticle interactions). So, at a first glance, time-independent simulations already suggest that anisotropy energy barriers and hence the switching time provided by Equation 5 may be altered by the volume fraction of the system of monodisperse (identical) nanoparticles. However, any information on the characteristic time constant τ_0_ is out of reach with this approach and this aspect will be investigated further by time-dependent simulations.

### Time-dependent approach: simulation and data analysis

**Figure 4:**
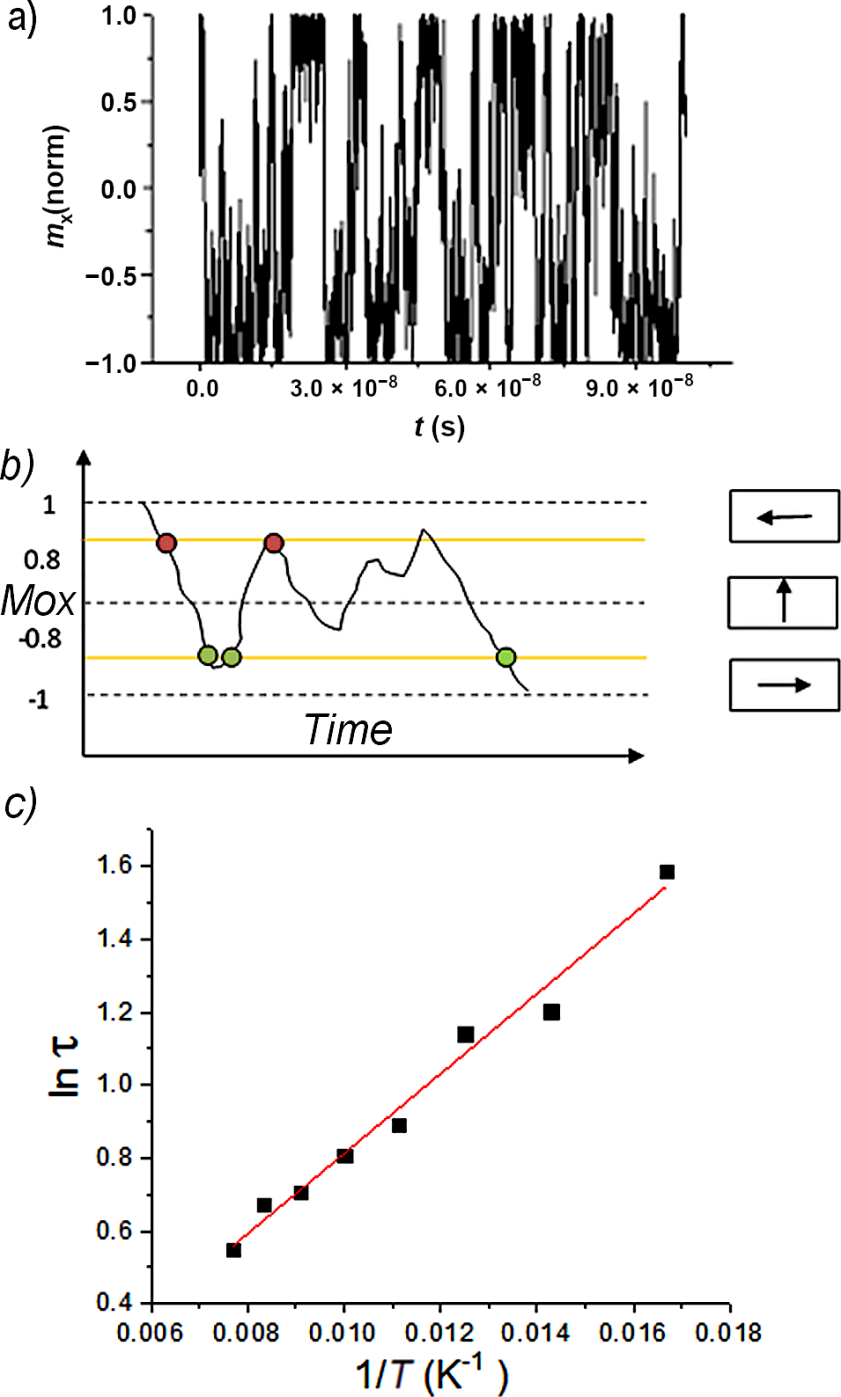
Temporal evolution of normalized magnetization along the *Ox* axis for particle P1 within a high volumetric fraction configuration, at 60 K (a). Counting method for the random switching of normalized magnetization (b). Arrhenius plot for the same particle, in the same configuration for temperatures ranging from 60 K to 140 K (c).

The magnetic switching of each nanoparticle in all three geometric configurations described above has been investigated via time-dependent (dynamic) micromagnetic simulations (numerical solution of Equation 7) performed at different temperatures (e.g., from 60 K to 140 K). The time dependence of the magnetic moment of one particle (e.g., P1 at 60 K) along the *Ox* axis (easy magnetization axis) is presented in Figure 4a. Large files consisting of such time dependences of the normalized magnetic moment (magnetization) have been obtained for each MNPs P1 at the mentioned temperatures and volume fractions. The following methodology was applied for a reliable evaluation of both the anisotropy energy barrier and relaxation time constant for any MNP of type P1, for a specific volume fraction.

The relaxation time, τ, was properly estimated (see Figure 4b) for each temperature from the time dependencies of the magnetic moment of P1 (similar to the one presented in Figure 4a). Then a plot of ln τ vs (1/*T*) according to Equation 5 was considered. Such an Arrhenius plot (logarithmic representation of Equation 5) is shown in Figure 4c for MNP P1, in the case of a high volume fraction configuration. The reasonable linear dependence suggests on one hand the validity of the perturbation-like treatment of the relaxation time even for the high concentration case, and on the other hand, it provides both the anisotropy energy barrier ΔE* and the relaxation time constant τ_0_* assumed to be affected by the interparticle interactions.

It is worth noting that the above-mentioned methodology depends critically on the reliability in the determination of the relaxation time (or switching frequency) from Figure 4a like dependencies. In this respect, a C^++^ program has been developed for automatically computing the number of magnetic switching events over a certain period (e.g., during 100 ns) from such time-dependent magnetization curves. Given a noise of 0.2 in Figure 4a like curves, any jump of the normalized magnetization between a maximum of 0.8 and a minimum of −0.8 was regarded as a switching event. Each intersection of the plots with the 0.8 and −0.8 lines triggers an *H* and *L* flag, respectively. A switching event is counted when *H* and *L* are both triggered. The schematic representation of the counting method is shown in Figure 4b. The relaxation time is provided by dividing the total simulation time by the total number of switching events.

**Figure 5:**
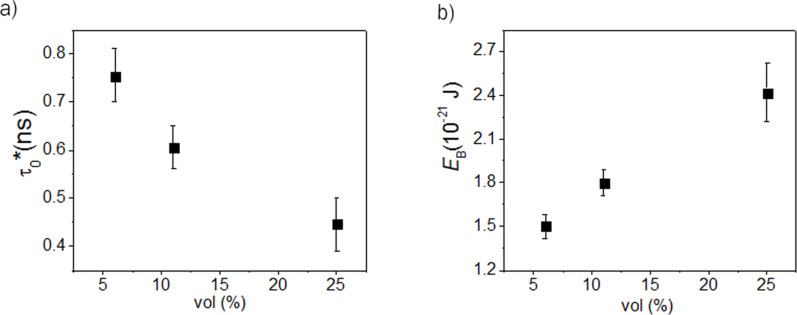
Relaxation time constant (a) and anisotropy energy barrier (b) of an inner MNP versus the volume fraction in %, as estimated from the theoretical simulations.

The values obtained for the anisotropy energy *E*_B_ = ΔE* and the time constant τ_0_* for the inner MNP P1 (considered as representative in assemblies of a large number of MNPs) for different volume fractions are shown in Figure 5.

It is worth noting that the equality between the anisotropy energy barrier per nanoparticle obtained in the case of static and dynamic (temperature dependent) simulations is not straightforward due to the fact that in the first case the external excitation (i.e., the magnetic field) has been applied strictly along the *Ox* axis whereas in the second case, the excitations, including the fluctuating field due to temperature activation of the surrounding magnetic moments (described by **h_fluct_**) act on all three axes. It should also be mentioned that both the anisotropy energy and relaxation time constant depend not only on the volume fraction (average distances between MNPs) but also on the number of magnetic neighbors, being different for outer or inner MNPs, but also for a bidimensional or tridimensional configurations of neighbors. Given the extension of a real system of MNPs relative to the present simulations, the most representative values describing the magnetic relaxation regime of ferrofluids are those corresponding to inner MNPs, as above mentioned, whereas the tridimensional arrangement (equivalent to an increased number of neighbors at a similar volume fraction and hence, to an increased interaction) would be reflected by an amplification of the specific behaviors observed from the bidimensional configuration of the MNPs. According to Figure 5, the relaxation time constant decreases almost exponentially and the anisotropy energy barrier of the nanoparticle increases almost linearly with the volume fraction. This result is in reasonable qualitative agreement with the experimental results presented in Figure 1.

## Conclusion

The direct implications of the magnetic relaxation phenomena of interacting superparamagnetic nanoparticles on the specific absorption rate of ferrofluids, through susceptibility loss mechanisms, are discussed. Various models supporting the influence of interparticle interactions on the relaxation time are reviewed, leading to the conclusion that they are controversial, not versatile enough, and are limited to particular cases. A more general perturbation approach of the simple Néel expression is introduced with both the relaxation time constant and the anisotropy energy barrier depending on interparticle interactions, as suggested by specific absorption rate measurements performed on ferrofluids of different volume fractions. A theoretical investigation of such parameters versus interparticle interactions was performed via static and time dependent micromagnetic simulations on different configurations of nanoparticles. It has been proven that a monotonous increase of the anisotropy energy barrier and a quasi-exponential decrease of the relaxation time constant versus interparticle interactions, even up to the highest experimentally reachable volume fractions of ferrofluids is present. The mentioned evolutions of the two parameters (anisotropy energy barrier and relaxation time constant) in relation to interparticle interactions lead to a significant decrease in the SAR values in ferrofluids of high volume fraction, which should be taken into account in hyperthermia therapy applications.
